# Type Three Secretion System in Attaching and Effacing Pathogens

**DOI:** 10.3389/fcimb.2016.00129

**Published:** 2016-10-21

**Authors:** Meztlli O. Gaytán, Verónica I. Martínez-Santos, Eduardo Soto, Bertha González-Pedrajo

**Affiliations:** Departamento de Genética Molecular, Instituto de Fisiología Celular, Universidad Nacional Autónoma de MéxicoCiudad de México, Mexico

**Keywords:** A/E pathogens, EPEC, EHEC, *Citrobacter rodentium*, locus of enterocyte effacement, type III secretion system, injectisome, secretion hierarchy

## Abstract

Enteropathogenic *Escherichia coli* and enterohemorrhagic *E. coli* are diarrheagenic bacterial human pathogens that cause severe gastroenteritis. These enteric pathotypes, together with the mouse pathogen *Citrobacter rodentium*, belong to the family of attaching and effacing pathogens that form a distinctive histological lesion in the intestinal epithelium. The virulence of these bacteria depends on a type III secretion system (T3SS), which mediates the translocation of effector proteins from the bacterial cytosol into the infected cells. The core architecture of the T3SS consists of a multi-ring basal body embedded in the bacterial membranes, a periplasmic inner rod, a transmembrane export apparatus in the inner membrane, and cytosolic components including an ATPase complex and the C-ring. In addition, two distinct hollow appendages are assembled on the extracellular face of the basal body creating a channel for protein secretion: an approximately 23 nm needle, and a filament that extends up to 600 nm. This filamentous structure allows these pathogens to get through the host cells mucus barrier. Upon contact with the target cell, a translocation pore is assembled in the host membrane through which the effector proteins are injected. Assembly of the T3SS is strictly regulated to ensure proper timing of substrate secretion. The different type III substrates coexist in the bacterial cytoplasm, and their hierarchical secretion is determined by specialized chaperones in coordination with two molecular switches and the so-called sorting platform. In this review, we present recent advances in the understanding of the T3SS in attaching and effacing pathogens.

## Introduction

The attaching and effacing (A/E) family of gastrointestinal bacterial pathogens induces a singular phenotype on host cells called the A/E lesion, characterized by the effacement of epithelial microvilli and the subsequent formation of actin-rich protruding structures known as pedestals right beneath the adherent bacteria, to which they remain intimately attached (Moon et al., 1983; Knutton et al., 1989; Nataro and Kaper, 1998). Members of this family include the human pathogens enteropathogenic *Escherichia coli* (EPEC) and enterohemorrhagic *E. coli* (EHEC), as well as the mouse pathogen *Citrobacter rodentium*, which is the representative organism used to understand the molecular basis of A/E lesion formation in an infection animal model (Goosney et al., 2000; Collins et al., 2014). EPEC is considered one of the predominant causative agents of human diarrhea in developing countries, affecting principally infants aged 0–11 months, representing an important cause of mortality. It causes moderate to severe protracted diarrhea, accompanied by mild fever and sometimes vomiting (Nataro and Kaper, 1998; Kotloff et al., 2013). EPEC strains can also colonize rabbits (REPEC; rabbit-EPEC), mimicking to some extent the colonization process in humans, so this organism has been used as a model for studying EPEC infection (Milon et al., 1999; Zhu et al., 2006). EHEC is an emerging zoonotic pathogen that can cause acute gastroenteritis and hemorrhagic colitis in children younger than 5 years and the elderly (Boyce et al., 1995). It can also colonize ruminants, especially cattle, without being pathogenic, but rather using them as reservoirs (Su and Brandt, 1995). In 1982, EHEC serotype O157:H7 was recognized as a human pathogen during two outbreaks associated with the ingestion of undercooked meat in the United States of America (Riley et al., 1983). In severe cases, due to the translocation of Shiga toxins (Stx1 and Stx2) across the gut, it can produce hemolytic uremic syndrome, which can lead to kidney failure and chronic post-infection sequelae or death (Frankel et al., 1998; Tarr et al., 2005; Spinale et al., 2013). Two main differences between these pathogens, besides the presence of Shiga toxins in EHEC, are the infectious dose and cellular tropism. While a dose between 10^8^ and 10^10^ bacteria is needed for EPEC to cause disease in adult volunteers (Donnenberg et al., 1993; Bieber et al., 1998), it has been estimated from EHEC found in contaminated food, that only 10–100 colony-forming units are sufficient for infection (Armstrong et al., 1996). With respect to tropism, EPEC colonizes the small intestine, specifically the duodenum, terminal ileum and Peyer's patches, while EHEC colonizes mainly the Peyer's patches and the large bowel (Phillips et al., 2000; Fitzhenry et al., 2002). Lastly, *C. rodentium* is a natural mouse pathogen that is genetically related to *E. coli* and forms A/E lesions in intestinal cells (Schauer and Falkow, 1993). Mice infected with *C. rodentium* develop transmissible murine colonic hyperplasia, a disease characterized by proliferation of epithelial colonic cells that can then turn into diarrhea (Mundy et al., 2005).

A/E lesion development occurs in three stages: (i) initial adherence, (ii) signal transduction, and (iii) intimate attachment (Donnenberg and Kaper, 1992). The first stage has been better characterized for EPEC, where it has been shown that bacteria adhere to host cells in a localized pattern through a type IV pilus (T4P) named BFP (bundle-forming pilus), which is also involved in bacterium-to-bacterium adherence (Girón et al., 1991). EHEC and *C. rodentium* also possess T4P, named HCP (hemorrhagic coli pilus) and CFC (colonization factor *Citrobacter*), respectively, that have been proposed to be involved in cell adherence and colonization (Mundy et al., 2003; Xicohtencatl-Cortes et al., 2007). Other fimbrial and afimbrial adhesins have been implicated in the initial adherence process such as intimin, flagella, and the *E. coli* common pilus, among others (Donnenberg and Kaper, 1991; Girón et al., 2002; Cleary et al., 2004; Rendón et al., 2007; Saldana et al., 2009). In the second stage, the bacteria interfere with a variety of signal transduction pathways in the host cell through the translocation of several virulence proteins, called effectors, via a highly conserved specialized protein-secretion apparatus called the type III secretion system (T3SS) (Garmendia et al., 2005). The number of translocated effectors varies from approximately 22 in EPEC and 29 in *C. rodentium*, to as many as 39 in certain EHEC strains (Tobe et al., 2006; Deng et al., 2010, 2012; Petty et al., 2010). Some changes induced by these proteins include modification of the host actin cytoskeleton (Campellone et al., 2004), failure of microtubule function (Hardwidge et al., 2005; Shaw et al., 2005; Tomson et al., 2005), inhibition of ion transport (Hodges et al., 2008), and disruption of epithelial barrier function (Viswanathan et al., 2009). In the third stage, the bacteria bind intimately to the host cell through an outer membrane protein called intimin, which functions as the ligand for the translocated receptor protein Tir inserted in the host cell membrane. The actin of microvilli surrounding attached bacteria is reabsorbed and the pedestal-like structure is completed leading to A/E lesion formation and disease (Jerse and Kaper, 1991; Kenny and Finlay, 1997; Kenny et al., 1997b). This tight adhesion to the host's epithelia would provide an advantage to the pathogen for outcompeting normal microbiota (Vallance and Finlay, 2000).

As aforementioned, the T3SS plays a crucial role in A/E lesion formation and is essential to the virulence of these bacteria. In recent years, a notable progress has been achieved in our understanding of the T3SS machinery in A/E pathogens. This review aims to explore the assembly, structure and function of the T3SS in this important family of pathogens, highlighting its major differences with archetypical systems from *Yersinia* spp., *Salmonella enterica*, and *Shigella flexneri*. Therefore, to allow a straightforward comparison between homologous proteins, the unified nomenclature Sct (secretion and cellular translocation; Hueck, 1998) will also be used throughout the review.

## Locus of enterocyte effacement

In A/E pathogens the T3SS is encoded by a ca. 35 kb chromosomally located pathogenicity island (PAI) named the locus of enterocyte effacement (LEE) (Jarvis et al., 1995). The LEE genes are conserved in all A/E pathogens, however it has been shown that, when introduced in a non-pathogenic *E. coli* K12 strain, the LEE of EPEC is sufficient for A/E lesion formation, whereas the LEE of EHEC is not (McDaniel and Kaper, 1997; Elliott et al., 1999b). The fact that the GC content (ca. 38%) of these PAIs is significantly lower than that of the average of *E. coli* and *C. rodentium* genomes (50.8 and 54.6%, respectively), reflects that they were acquired by horizontal gene transfer (Frankel et al., 1998; Deng et al., 2001; Schmidt and Hensel, 2004).

**Figure 1:**
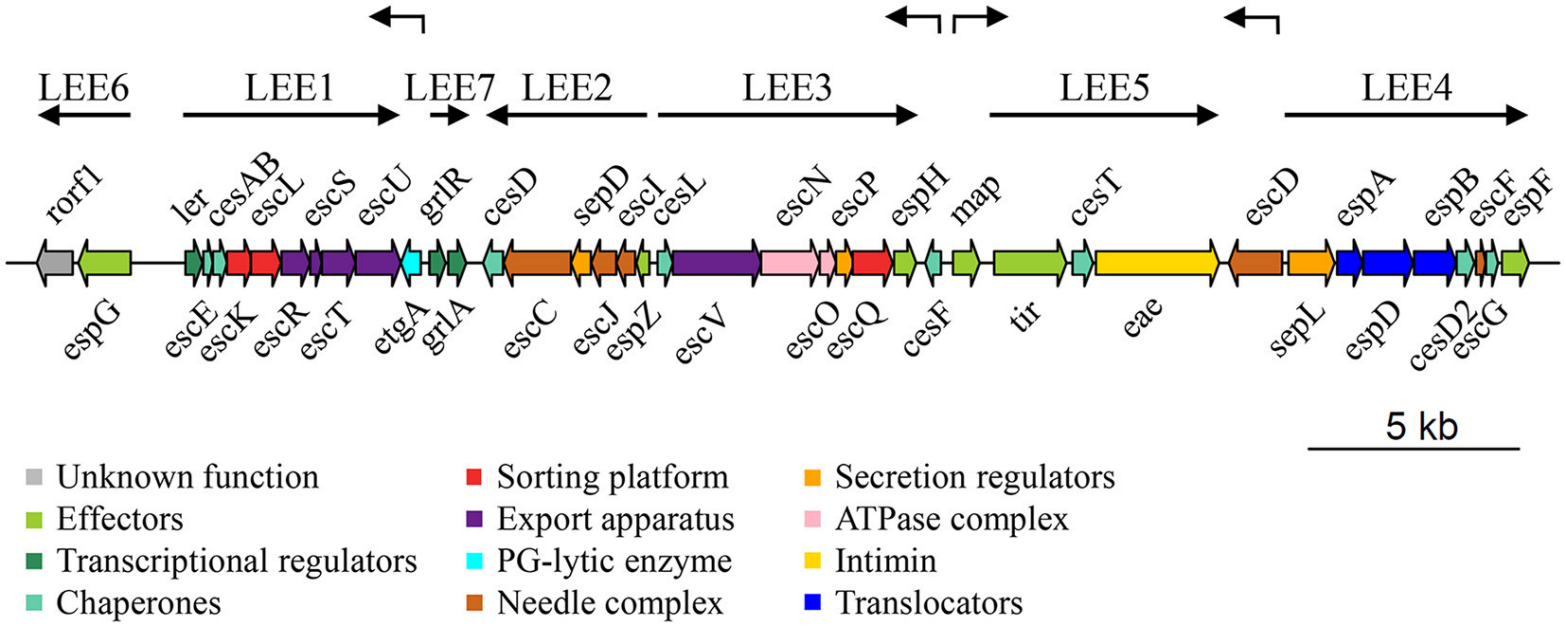
**Genetic structure of the LEE island of *Escherichia coli* E2348/69 O127:H6 strain (EPEC)**. Genes are depicted as filled arrows colored according to their proposed functional category (see enclosed box). The operon organization of the island (LEE1 to LEE7) is indicated by solid black arrows above the LEE genes, while individual transcriptional units (*etgA, cesF, map, escD*) are denoted by broken arrows. Schematic representation is drawn at scale (scale bar 5 kb). PG, peptidoglycan.

McDaniel and colleagues described the LEE for the first time in EPEC E2348/69 (McDaniel et al., 1995). It contains 41 genes organized in seven operons (LEE1 to LEE7) and four monocistronic units, as illustrated in Figure 1 (Elliott et al., 1998; Mellies et al., 1999; Sánchez-SanMartin et al., 2001; Barba et al., 2005; Yerushalmi et al., 2014). The LEE encodes all the structural components of the T3SS, seven of the effectors translocated through this system and their cognate chaperones, proteins involved in bacterial intimate adherence, and proteins that participate in secretion regulation and LEE expression (Table 1). All the genes encoded in the LEE of EPEC are present in the LEE of EHEC in the same order, and have a high degree of conservation showing an average identity of 94% at the nucleotide level (Perna et al., 1998). The LEE PAI is also conserved in *C. rodentium*, but with some differences with respect to that of EPEC and EHEC. The LEE islands of EPEC and EHEC are inserted into the selenocysteine tRNA locus; while the *C. rodentium* LEE is inserted in a different chromosomal location flanked by an IS element followed by an operon encoding an ABC transport system and plasmid sequences. Furthermore, the LEE6 operon of *C. rodentium*, containing the *rorf1* and *espG* genes, is positioned at the opposite end of the LEE with respect to EPEC and EHEC (Deng et al., 2001; Petty et al., 2010).

Table 1**Functional organization of the LEE encoded T3SS components**.

**Table:** 

**Extracellular components**				**PG-lytic enzyme**	**Adhesin**	**Basal body**								
**Translocation pore**		**Filament**	**Needle**			**IM ring**		**OM ring**	**Inner rod**	**Export apparatus**				
EspB	EspD	EspA	EscF	EtgA	Intimin	EscD	EscJ	EscD	EscI	EscR	EscS	EscT	EscU	EscV

**Table:** 

**Cytoplasmic components**																		
**ATPase complex**					**Secretion regulation**			**Transcriptional regulators**			**Chaperones**							
		**Sorting platform**									**Early substrates**		**Middle substrates**			**Late substrates**		
EscN	EscO	EscL	EscQ	EscK	EscP	SepL	SepD	Ler	GrlA	GrlR	EscE	EscG	CesAB	CesD	CesD2	CesT	CesF	CesL

**Table:** 

**Cytoplasmic components**						
**LEE-encoded effectors**						
EspG	EspZ	EspH	Map	Tir	EspF	EspB

*Proteins encoded in the LEE are ordered according to their assigned function. EscL and EspB are classified into two different categories: EscL as a sorting platform and ATPase complex component, and EspB as a translocator and effector*.

The proper expression of the LEE is a complex process that depends on several factors, such as environmental conditions, quorum sensing, and transcriptional as well as post-transcriptional regulation (Connolly et al., 2015). The LEE encodes its own transcriptional regulators, named Ler, GrlA, and GrlR (Mellies et al., 1999; Deng et al., 2004). Ler is a 15-kDa protein encoded by the first gene of the LEE1 operon that acts as the central regulator of LEE gene expression (Mellies et al., 1999). This protein belongs to the H-NS-like protein family whose main representative, H-NS, negatively regulates the expression of several horizontally acquired genetic elements, including the LEE (Bustamante et al., 2001; Umanski et al., 2002; Dorman, 2004). Ler counteracts the repression exerted by H-NS and thus is essential for the expression of the LEE (Bustamante et al., 2001; Winardhi et al., 2014). Interestingly, Ler also acts as a negative regulator of its own expression (Berdichevsky et al., 2005; Bhat et al., 2014). In addition, GrlA and GrlR, encoded in the bicistronic LEE7 operon, positively and negatively regulate Ler expression (Deng et al., 2004; Huang and Syu, 2008). GrlA has been shown to activate LEE gene expression through its direct binding to the LEE1 promoter (Jiménez et al., 2010), and Ler controls the expression of the LEE7 operon, thus forming a positive regulatory loop (Barba et al., 2005). On the other hand, GrlR represses LEE gene expression, although the mechanism of repression is still unknown. GrlR has been demonstrated to interact directly with GrlA (Creasey et al., 2003b; Padavannil et al., 2013); therefore, it was proposed that GrlR represses LEE transcription by sequestering GrlA, avoiding its binding to the LEE1 promoter and thus repressing *ler* expression (Huang and Syu, 2008).

Besides these three transcriptional regulators, a fourth LEE-encoded protein has been proposed to modulate LEE expression in EHEC (Sun et al., 2016). The Mpc (multiple point controller) protein, also known as CesL (previously Orf12 or L0036), was shown to interact with Ler (Tsai et al., 2006; Younis et al., 2010). When Mpc is overexpressed, it sequesters Ler impeding its function and leading to the repression of the LEE encoded genes (Tsai et al., 2006). Furthermore, in typical EPEC strains containing the EAF (EPEC adherence factor) plasmid, LEE expression is also positively regulated by PerC, a transcriptional regulator encoded by the *perABC* plasmid-located operon (Gómez-Duarte and Kaper, 1995; Tobe et al., 1996). EHEC possess three PerC homologous proteins named PchA, PchB and PchC (Iyoda and Watanabe, 2004), while there is no known PerC homolog in *C. rodentium*.

To illustrate the complexity of the regulatory networks that govern LEE expression suffice it to mention that several global transcriptional regulators are involved in LEE regulation, such as BipA, Cpx, Fis, GadE, Hha, H-NS, H-NST, IHF, RgdR, RpoN, SspA, FusK/R, EutR, LeuO, SdiA, KdpE, QseA, QseC, QseD, QseE, RcsB, RegA, and GlmY/GlmZ. Besides, LEE expression is also regulated at the post-transcriptional level by ClpXP, CsrA, DegP, DsrA, Hfq, RNaseE, and RpoS, reviewed in Deane et al. (2010), Yang et al. (2010) Levine et al. (2014) and Franzin and Sircili (2015).

## Structure of the type III secretion system

**Figure 2:**
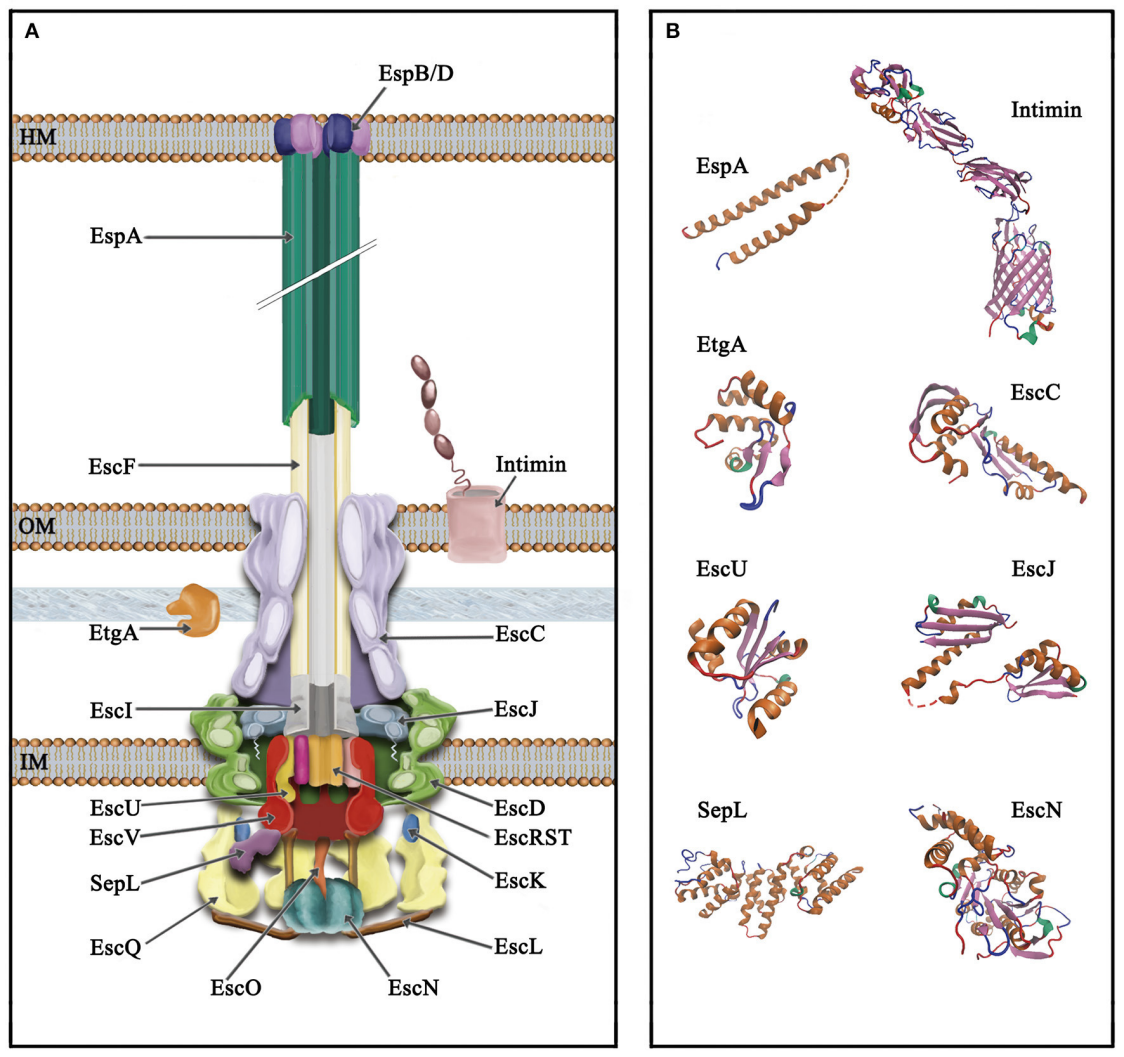
**Schematic representation of the type III secretion system of A/E pathogens. (A)** The T3SS is divided into three main parts, from top to bottom (i) extracellular appendages: translocation pore (inserted into the host membrane, HM), filament and needle; (ii) basal body: consisting of three membrane rings that span the inner and outer membrane (IM and OM, respectively) connected through a periplasmic inner rod. The IM rings house the export apparatus components; (iii) cytoplasmic components: the C-ring, the ATPase complex and the gatekeeper protein. The outer membrane protein intimin and the PG lytic enzyme EtgA are also illustrated. **(B)** Solved protein structures of the depicted T3SS components. Protein Data Bank (PDB) accession numbers: SepL, 5C9E; EscN, 2OBM; cytoplasmic C-terminal domain of EscU, 3BZL; periplasmic domain of EscC, 3GR5; EtgA, 4XP8; periplasmic domain of EscJ, 1YJ7; the EspA structure was obtained from that of the CesAB/EspA complex, 1XOU, chain A; transmembrane beta-domain of intimin, 4E1S and its C-terminal domain, 1F00. Protein structures are displayed as ribbon diagrams and were colored according to their secondary structure.

The T3SS or injectisome is a complex nanomolecular machine of about 3.5 MDa consisting of more than 20 proteins. The injectisome global architecture is conserved among different bacterial species and resembles that of the evolutionarily related flagellar system (Hueck, 1998; Erhardt et al., 2010b; Abby and Rocha, 2012). It is composed of a syringe shaped-structure protruding above the bacterial surface with a central channel of 2–3 nm in diameter, and three ring structures embedded in the inner and outer bacterial membranes, connected through a periplasmic inner rod (Hueck, 1998; Burkinshaw and Strynadka, 2014; Notti and Stebbins, 2016). Although, the core architecture of this so-called needle complex is highly similar in bacteria analyzed so far, the supramolecular structure of the EPEC and EHEC T3SS shows an extracellular filament assembled on top of the needle (see below; Ebel et al., 1998; Knutton et al., 1998; Daniell et al., 2001b; Sekiya et al., 2001). Overall, the components of the T3SS can be grouped according to the substructures they form, from outside in: extracellular appendages, basal body and cytoplasmic components. A schematic representation of the T3SS in A/E pathogens as well as several solved protein structures are depicted in Figure 2.

### Extracellular appendages

#### The needle

The needle is a superhelical hollow structure comprised of multiple copies of the EscF protein (Wilson et al., 2001), which is essential for the secretion of all T3 substrates and hence for virulence (Deng et al., 2004). EscF associates with the membrane ring proteins EscC, EscD, and EscJ (Ogino et al., 2006), as well as with the filament protein EspA (Wilson et al., 2001), forming a continuous channel that connects the bacterial cytoplasm with the host cell (Figure 2). It also associates in the cytoplasm with two chaperones, EscE and EscG that prevent its premature polymerization and are essential for its assembly (Sal-Man et al., 2013).

In EPEC, the needle is 23 nm in length and 8–9 nm in width (Sekiya et al., 2001; Ogino et al., 2006; Monjarás Feria et al., 2012). Although, the inner diameter of the needle central conduit in A/E pathogens has not been determined, in other T3S systems it ranges from about 1.3 to 2.5 nm (Blocker et al., 2001; Fujii et al., 2012; Loquet et al., 2012). The size of this channel is not wide enough to accommodate folded proteins; therefore, effectors should be unfolded prior to transport through to the T3SS (Feldman et al., 2002; Akeda and Galán, 2005; Fujii et al., 2012). This hypothesis was demonstrated by the fusion of a stable protein, or bulky proteins containing a knotted motif, to the C-terminal ends of T3 substrates. The fusion proteins were trapped inside the needle, blocking further secretion and allowing its co-purification with isolated T3S needle complexes (Dohlich et al., 2014; Radics et al., 2014). Visualization of the trapped recombinant substrates by cryo-EM provided direct evidence for the passage of unfolded proteins through this structure (Radics et al., 2014).

The EPEC needle structure is one of the smallest characterized, compared to those of *S. enterica* (25–80 nm) (Kubori et al., 1998; Kimbrough and Miller, 2000; Marlovits et al., 2006), *S. flexneri* (45–50 nm) (Tamano et al., 2000), *Y. pestis* (41 nm), and *Y. enterocolitica* (58 nm) (Journet et al., 2003). The needle length is strictly regulated by and correlates with the size of a family of proteins named type III secretion substrate specificity switch (T3S4) proteins (Journet et al., 2003; Büttner, 2012). In A/E pathogens, the EscP protein belongs to the T3S4 protein family, and in EPEC it was demonstrated to directly interact with EscF (Monjarás Feria et al., 2012). This interaction is important for needle length regulation in a process that will be discussed below. Furthermore, in other bacteria it has been proposed that the needle plays an active role in mammalian cell sensing and substrate secretion regulation (Kenjale et al., 2005; Torruellas et al., 2005); however, no direct evidence about this process has been reported in A/E pathogens.

Remarkably, the number of needle complexes in EPEC has been estimated to be 12 per cell, based on the observed EspA filaments (Daniell et al., 2001b; Wilson et al., 2001), which is fewer than the number seen in *Salmonella* (10–100; Kubori et al., 1998) and *Yersinia* (30–100; Hoiczyk and Blobel, 2001).

#### The filament

The filament is an extracellular appendage found in the T3S systems of A/E pathogens (also present in *Bordetella* spp.) that functions as an adaptor between the needle and the translocation pore formed in the host cell membrane (Figure 2; Knutton et al., 1998; Ide et al., 2001; Medhekar et al., 2009). In A/E pathogens, it is assembled by the polymerization of multiple subunits of the EspA protein with a helical symmetry of 5.6 subunits per turn (Knutton et al., 1998; Daniell et al., 2003; Wang et al., 2006), forming a hollow structure of 12 nm in width that allows the passage of substrates through a 2.5 nm central channel (Daniell et al., 2001b, 2003; Crepin et al., 2005b). Unlike the needle, the EspA filament has a variable length that can reach more than 600 nm, which seems to be dependent on the availability of EspA subunits given that it can be enlarged by increasing the amount of EspA in the cytoplasm (Sekiya et al., 2001; Crepin et al., 2005b). The average length of the filament is ca. 90 nm and it elongates by addition of EspA subunits at the tip of the structure (Daniell et al., 2003; Crepin et al., 2005b).

The EspA protein has a coiled-coil domain at its C-terminal region that is required for subunit polymerization and assembly of the filament (Delahay et al., 1999). Since this protein undergoes spontaneous polymerization, it requires the assistance of chaperone proteins in the cytoplasm. CesAB was the first EspA chaperone to be described (Creasey et al., 2003c). The crystal structure of the CesAB-EspA complex showed that the coiled-coil domain of EspA is also the one involved in the interaction with CesAB, thus revealing the mechanism that prevents EspA premature oligomerization (Yip et al., 2005a). Besides CesAB, EspA has a second chaperone named CesA2 (formerly Orf29, renamed EscG in EPEC) that assists EspA stabilization in the cytoplasm (Su et al., 2008). In EHEC it has also been reported that EspA binds to EscL, a component of the ATPase complex, and that this interaction is required to preserve EspA stability, although not via a chaperoning mechanism (Ku et al., 2009). EspA can also directly bind to EscF and EspB, forming a continuous channel from the bacterial cytoplasm to the host cell cytosol (Hartland et al., 2000; Daniell et al., 2001b).

Apart from its role as a translocation conduit, and similarly to the needle, the EspA filament has been proposed to participate in sensing the presence of mammalian cells, and there is evidence that involves this structure in adhesion to epithelial cells and biofilm formation (Ebel et al., 1998; Knutton et al., 1998; Cleary et al., 2004; Moreira et al., 2006). However, the EspA filaments are not present over the entire course of infection; once the intimate attachment has been established, they are eliminated and so are absent from the mature A/E lesion. Indeed, it has been reported that expression of the *espA* gene, and thus the presence of EspA filaments, is downregulated after 6 h of infection (Knutton et al., 1998; Dahan et al., 2004).

#### The translocation pore

The translocation pore is a protein complex formed by the hetero-oligomerization of EspB and EspD subunits. These proteins interact with each other and insert into the host membrane, forming a channel that allows the direct translocation of effectors from the bacteria to the host cell cytoplasm (Figure 2; Ide et al., 2001). The EspB and EspD proteins are predicted to have one and two transmembrane domains, respectively, involved in their membrane anchoring (Delahay and Frankel, 2002; Dasanayake et al., 2011). It has been shown that they can insert into erythrocyte membranes causing red blood cells (RBCs) hemolysis (Warawa et al., 1999). Moreover, both of these hydrophobic translocators interact with the hydrophilic translocator EspA, forming the so-called translocon (Hartland et al., 2000; Luo and Donnenberg, 2011). Mutants in any of the translocon components are still able to secrete proteins to the medium, but fail to translocate them to the host cell, thus impairing adhesion, A/E lesion formation and virulence (Lai et al., 1997; Kresse et al., 1999; Deng et al., 2004).

Analysis with low resolution atomic force microscopy showed that the pores formed by EspB and EspD in diffusely adhering EPEC (DA-EPEC) are composed of six to eight subunits with a minimal pore size of 3–5 nm (Ide et al., 2001). In addition, EspD, which is able to interact with itself through a C-terminal coiled-coil domain (Daniell et al., 2001a), spontaneously incorporates into unilamellar vesicles, forming a pore with an inner diameter of 2.5 nm and a molecular mass of 280–320 kDa, which would consist of six to seven subunits (Chatterjee et al., 2015). In agreement with this, analysis of RBC membranes during EPEC-mediated hemolysis showed that EspD was the only bacterial protein membrane-associated, suggesting it plays a dominant role in pore formation. In contrast, EspB seems to have a secondary role in pore formation since a Δ*espB* mutant caused only a slight reduction in hemolysis (Shaw et al., 2001).

Although, significant advances have been made in determining the components that form the translocation pore, and to a lesser extent, its stoichiometry, little is known about their topology when inserted into the host membrane. For *P. aeruginosa* translocator proteins PopB and PopD (EspD and EspB homologs, respectively), it has been shown that PopB is inserted into the host membrane with both the N- and C-terminus facing the outer leaflet of host plasma membrane. Likewise, the C-terminus of PopD is located toward the extracellular milieu, where it docks to the needle tip formed by PcrV (EspA in A/E pathogens), while its N-terminus is facing the host cytoplasm (Discola et al., 2014; Armentrout and Rietsch, 2016). The proposed membrane orientation of PopD differs from that reported for EspB in EPEC where, by introducing sequences recognized by a host kinase, it was demonstrated that the C-terminal domain of EspB is localized in the host cytoplasm while the N-terminal domain remains extracellular (Luo and Donnenberg, 2011).

Additionally, it has been reported that cholesterol membrane content plays an important role in EPEC and EHEC adherence, protein translocation and pedestal formation (Hayward et al., 2005; Riff et al., 2005). In fact, it was shown that the EspD homologs in *Salmonella* (SipB) and *Shigella* (IpaB) bind with high affinity to cholesterol, which is required for efficient delivery of effectors into host cells (Hayward et al., 2005). However, even though it was initially reported that EspD inserts into Triton X-100 resistant membrane domains (Wachter et al., 1999), and binds to vesicles with a lipid composition that resembles that of the eukaryotic outer leaflet of the membrane, it was later demonstrated that cholesterol is not required for EspD binding or pore formation; instead, addition of anionic lipids such as phosphatidylserine or phosphaditylglycerol induces pore formation of EspD in unilamellar vesicles (Chatterjee et al., 2015). It is possible that cholesterol is necessary for the function of effectors associated with the host membrane rather than for the translocon assembly *per se* (Allen-Vercoe et al., 2006; Chatterjee et al., 2015); however, this issue remains a matter of debate. Recently, the translocon pore-forming activity was demonstrated to be regulated by the serine protease autotransporters EspC and EspP in EPEC and EHEC, respectively (Guignot et al., 2015). EspC degrades EspA and EspD after host cell contact, regulating the translocation pore forming activity, which in turn downregulates the cytotoxicity induced by EPEC (Guignot et al., 2015). This is in agreement with previous findings showing that EspC interacts with EspA (Vidal and Navarro-García, 2008) and that a mutant defective in the EspC ortholog in *C. rodentium* displayed increased virulence in an *in vivo* infection model (Vijayakumar et al., 2014).

Besides its role as a pore-forming component, EspB is as well translocated into host cells, where it binds to myosin, inhibiting its interaction with actin and contributing to microvilli effacement and EPEC phagocytosis inhibition (Taylor et al., 1998; Wolff et al., 1998; Iizumi et al., 2007). It has also been demonstrated to bind and recruit α-catenin at the EHEC adherence site, helping to the development of the A/E lesion (Kodama et al., 2002). EspD and EspB are assisted in the bacterial cytoplasm by three chaperones CesD, CesD2, and CesAB that maintain them in an unfolded state and prevent their premature oligomerization (Wainwright and Kaper, 1998; Creasey et al., 2003c; Neves et al., 2003).

### Basal body

The basal body consists of three membrane rings connected through a periplasmic inner rod. The proteins EscD and EscJ form two concentric rings in the inner membrane (IM) and the EscC protein forms the outer membrane (OM) ring (Figure 2). The estimated values for the external widths of the OM and IM rings are 16.7 ± 1.9 nm and 18.1 ± 2.5 nm, respectively (Sekiya et al., 2001). These ring dimensions are similar to those reported for *Shigella* (15 ± 1.3 nm and 26.1 ± 1.3 nm; Tamano et al., 2000), and *Y. enterocolitica* (12 and 18 nm; Kudryashev et al., 2013). The height of the basal body, 31.4 ± 4.3 nm in EPEC and 31.6 ± 0.3 nm in *Shigella*, is also quite similar and presumably sufficient to traverse the bacterial membranes and the peptidoglycan layer (Sekiya et al., 2001).

**Figure 3:**
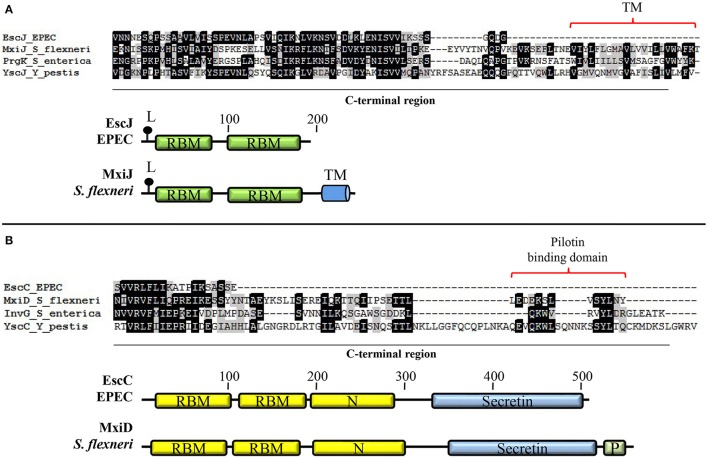
**Basal body components of A/E pathogens possess distinctive features**. **(A)** EscJ lacks the C-terminal transmembrane segment typically present in proteins of the SctJ family. Multiple protein alignment of the C-terminal domain of the lipoproteins EscJ (EPEC), MxiJ (*S. flexneri*), PrgK (*S. enterica*), and YscJ (*Y. pestis*) forming the inner membrane ring. The schematic comparison of EscJ and MxiJ domain organization is shown at the bottom. **(B)** The proposed pilotin-binding domain is absent in the EscC secretin. Multiple alignment of the C-terminal domain of secretins EscC (EPEC), MxiD (*S. flexneri*), InvG (*S. enterica*), and YscC (*Y. pestis*). The schematic comparison of EscC and MxiD domain organization is shown at the bottom. Even though only the basal body proteins from EPEC are depicted, all A/E pathogens share these features. TM, transmembrane helix; L, lipidation site; RBM, ring-building motif; Secretin, secretin domain (PF00263); N, secretin N domain (PF03958); P, pilotin-binding domain.

EscD possesses a predicted transmembrane domain (amino acids 120–141) as well as seven predicted myristoylation sites (Kresse et al., 1998; Ogino et al., 2006). Like its *Yersinia* homolog YscD, it contains a cytoplasmic forkhead associated (FHA) domain, which mediates phosphoprotein-recognition, and at least one putative phospholipid-binding domain (BON; bacterial OsmY and nodulation domain) in its periplasmic portion (Pallen et al., 2002, 2005; Gamez et al., 2012). The FHA domain of *Y. pestis* YscD is essential for T3SS formation and that of *S. flexneri* MxiG interacts with phosphorylated Spa33 (the EscQ homolog; Ross and Plano, 2011; Barison et al., 2012); however, its role in EscD function remains to be determined. The EscD protein associates with the OM ring protein EscC and the needle component EscF; yet, no direct interaction with the IM ring protein EscJ has been demonstrated (Creasey et al., 2003b; Ogino et al., 2006). Nevertheless, by analogy to its homologs in other systems (Kimbrough and Miller, 2000; Blocker et al., 2001; Schraidt et al., 2010), EscD is believed to form the outermost IM ring that surrounds the EscJ ring. EscJ is produced as a pre-protein and is presumed to be translocated to the periplasm in a Sec-dependent manner (Crepin et al., 2005a). Crystal packing analysis of the solved structure of this protein showed that it forms a 24 subunit ring of 18 nm width and 5.2 nm height that fits the dimensions of the IM rings observed by transmission electron microscopy (TEM) (Sekiya et al., 2001; Yip et al., 2005b). Unlike its homologs, EscJ does not possess a transmembrane helix in its C-terminal domain; instead, it is anchored to the outer leaflet of the IM through an N-terminal lipid modification site (Figure 3A). Hence, EscJ forms a periplasmic ring positioned on top of the IM rather than within it (Crepin et al., 2005a; Yip et al., 2005b). The EscJ flagellar homolog FliF, also self-assembles in an annular structure in the IM forming the MS (membrane and supramembrane) ring of the flagellar T3SS (Ueno et al., 1992; Bergeron, 2016). In contrast to its virulence counterparts, FliF contains two transmembrane helices and a C-terminal cytoplasmic domain that directly interacts with the C-ring component FliG (Marykwas et al., 1996; Levenson et al., 2012).

The OM ring component EscC is essential for T3 secretion and needle complex formation (Gauthier et al., 2003; Deng et al., 2004; Ogino et al., 2006). EscC belongs to the secretin family of proteins that function as channels for secretion of bacterial proteins across the OM in several secretion systems. They consist of two major domains: an N-terminal periplasmic region and a highly conserved C-terminal region embedded in the outer membrane (Genin and Boucher, 1994). EscC possesses a signal sequence that is cleaved after its Sec-dependent export across the IM. In many T3S systems, the oligomerization of the secretin and its insertion in the OM is promoted by a lipoprotein called pilotin (Crago and Koronakis, 1998; Schuch and Maurelli, 2001; Burghout et al., 2004). However, this protein has not been identified in the T3SS of A/E pathogens and, in agreement, the C-terminal pilotin binding domain is absent in the EscC protein of EPEC, as shown in Figure 3B. In this regard, it has been reported that the proper OM localization of EscC requires the aid of other T3 apparatus components (Gauthier et al., 2003).

The crystal structure of the periplasmic region of the secretin EscC (residues 21–174) revealed a modular architecture of two small domains connected by a linker region that has a similar fold to the IM proteins EscJ and PrgH (EscD homolog in *Salmonella*). This suggests that the conserved fold might provide a common ring assembly motif for oligomerization of OM and IM rings in T3S systems (Spreter et al., 2009). EscC oligomerizes into a 17 nm diameter ring (Ogino et al., 2006). The cryo-EM of InvG (EscC homolog in *Salmonella*) suggests that the OM ring is composed of 15 subunits (Schraidt and Marlovits, 2011; Bergeron et al., 2013), while the cryo-EM and scanning-TEM analysis of *Yersinia* YscC and *Shigella* MxiD indicate that these secretins are composed of 12 subunits (Hodgkinson et al., 2009; Kowal et al., 2013). In the case of EscC, Spreter and colleagues built 12- and 14-subunit ring models, which were then docked to the cryo-EM maps of the *S. enterica* T3SS basal body (Marlovits et al., 2004). Although both models fitted the density maps, there is currently more evidence favoring the 12-mer model (Spreter et al., 2009), anyhow the OM ring stoichiometry in the T3SS of A/E pathogens remains to be demonstrated.

Regarding EscC protein interactions it has been demonstrated that it associates with the IM ring EscD (Creasey et al., 2003b; Ogino et al., 2006) and with injectisome axial components like the inner rod EscI and the needle EscF (Creasey et al., 2003b; Sal-Man et al., 2012b). In addition, it has been shown that EscC interacts with EscO (previously EscA; Sal-Man et al., 2012a), an ATPase complex component (Romo-Castillo et al., 2014), yet the functional relevance of this interaction remains to be established.

It is presumed that the OM and IM rings are connected through a periplasmic inner rod formed by the oligomerization of the EscI protein (Pallen et al., 2005; Sal-Man et al., 2012b). When visualized by EM the inner rod appeared to have a small ring-like structure at the base (10 nm in diameter), and a stem portion of 9 nm in width which is identical to the diameter of the needle; the entire length of the rod is approximately 20 nm (Ogino et al., 2006). In contrast, a recent study of the inner rod in *S. enterica* showed that this structure is made of only one helical turn composed of six PrgJ subunits (Zilkenat et al., 2016).

Additionally, the EscI homologs in *Yersinia* and *Salmonella* have been shown to be involved in the process that regulates T3 substrate secretion (Marlovits et al., 2006; Wood et al., 2008; Lefebre and Galán, 2014). In agreement, in EPEC it has been demonstrated that EscI interacts with EscU (Sal-Man et al., 2012b) and with EscP (Monjarás Feria et al., 2012), components involved in substrate secretion regulation as will be discussed below.

Injectisome assembly faces a physical barrier; it must traverse the peptidoglycan (PG) layer. In EPEC this task is accomplished by the use of a specialized PG degrading enzyme named EtgA (Pallen et al., 2005) that localizes to the periplasm to exert its PG lytic activity and which is required for efficient T3SS assembly (García-Gómez et al., 2011). The recently solved structure of EtgA disclosed similarities in its active site with both lytic transglycosylases and lysozyme (Burkinshaw et al., 2015a). This enzyme has been shown to interact directly with the inner rod subunit EscI (Creasey et al., 2003b; Burkinshaw et al., 2015a). Moreover, the enzymatic activity of EtgA is enhanced in the presence of EscI, suggesting that this interaction not only spatially restricts the activity of EtgA but also stimulates it (Burkinshaw et al., 2015a).

### Export apparatus

One of the most conserved components of the injectisome is the export apparatus. This membrane-embedded complex is essential for T3S function and, in A/E pathogens, is built of five different proteins: EscR, EscS, EscT, EscU, and EscV (EscRSTUV; Deng et al., 2004; Diepold and Wagner, 2014; Portaliou et al., 2016). These five polytopic proteins, which have been annotated by sequence comparison to its homologs in *Yersinia* (Elliott et al., 1998; Pallen et al., 2005), are presumed to assemble in a patch of the cytoplasmic membrane enclosed by the two inner membrane rings of the basal body (Figure 2; Yip et al., 2005b; Moraes et al., 2008; Wagner et al., 2010a).

Apart from its requirement for T3 protein secretion, little is known about the integral membrane components of the export apparatus, although considerable information has also been obtained from the homologous proteins in the flagellar T3SS (Minamino, 2014). EscU and EscV, as well as their respective homologs, which possess large cytoplasmic domains, are the most studied components of this protein complex. EscU belongs to the YscU/FlhB family of proteins, whose members are predicted to have two major domains connected through a conserved flexible linker: an N-terminal domain of ca. 200 amino acids that contains four predicted transmembrane regions, and a cytoplasmic C-terminal domain of ca. 100 amino acids that undergoes autocleavage in its NPTH amino acid motif. This autoproteolytic event between the asparagine and proline residues has been proposed to participate in substrate specificity switching (discussed below; Lavander et al., 2002; Fraser et al., 2003; Ferris et al., 2005; Sorg et al., 2007; Deane et al., 2008a; Zarivach et al., 2008; Björnfot et al., 2009; Lountos et al., 2009; Smith et al., 2009; Wiesand et al., 2009; Lorenz and Büttner, 2011; Thomassin et al., 2011). EscU interacts directly with EscP and EscI, and both interactions have also been implicated in the switching event (Zarivach et al., 2008; Thomassin et al., 2011; Monjarás Feria et al., 2012; Sal-Man et al., 2012b).

EscV, the major constituent of the T3 export apparatus, is also composed of two main domains: an N-terminal region with eight transmembrane helices and a C-terminal cytoplasmic domain of ca. 340 amino acids, which is much larger than that of EscU (Ghosh, 2004; Moraes et al., 2008). MxiA, its homolog in *Shigella*, assembles into a homononameric ring that aligns with the secretion channel at the base of the inner membrane machinery (Abrusci et al., 2013). The cytoplasmic domain of several flagellar and virulence EscV homologs has been crystallized, showing that the proteins of the FlhA/YscV family conserve a structural fold of four subdomains, some of which participate in the self-association of the monomers that form the nonameric export gate ring (Bange et al., 2010; Lilic et al., 2010; Moore and Jia, 2010; Saijo-Hamano et al., 2010; Worrall et al., 2010). Furthermore, it has been shown that members of this protein family such as FlhA and HrcV (the EscV homolog in *Xanthomonas campestris*) interact with different T3S substrates, suggesting that they might play a role in substrate recognition (Bange et al., 2010; Minamino et al., 2012; Hartmann and Buttner, 2013; Kinoshita et al., 2013). In addition, both virulence and flagellar EscV homologs have been implicated in energy conversion from proton-motive force (pmf) into protein export work (Hara et al., 2011; Minamino et al., 2011; Lee et al., 2014). In agreement, it has been proposed that members of the SctV family could actually form a proton channel for energy transduction, coupling proton flow to the T3 secretion process (Minamino et al., 2011; Lee and Rietsch, 2015).

Altogether, considering what has been reported for the homologous proteins, the membrane components of the export apparatus EscRSTUV would contribute to the recruitment and regulation of initial insertion of substrates into the injectisome. Specifically, the C-terminal domains of the flagellar EscV and EscU homologs form a transmembrane export gate that controls substrate access to the T3SS central channel (Minamino and Namba, 2008; Minamino et al., 2010). Regarding the stoichiometry of these membrane components, it was recently demonstrated that the *Salmonella* SPI-1 export apparatus is composed of 5 SpaP (EscR), 1 SpaQ (EscS), 1 SpaR (EscT), 1 SpaS (EscU), and 9 InvA (EscV) subunits (Zilkenat et al., 2016). The export apparatus components EscRSTU are encoded in the LEE1 operon whose expression is activated immediately after T3SS induction, while the expression of other LEE operons is activated 70 min after induction (Yerushalmi et al., 2014). In the SPI-1 encoded *Salmonella* T3SS, the EscRST homologs (SpaPQR) are necessary for the recruitment and assembly of the remaining components of the needle complex (Wagner et al., 2010a). However, in the case of EPEC, interfering with the timing of expression of the LEE1 operon barely affected the T3SS assembly efficiency (Yerushalmi et al., 2014).

### Cytoplasmic components

#### ATPase complex

The ATPase complex is formed by the EscN, EscL, and EscO proteins (Figure 2), all of which are important for T3SS function (Deng et al., 2004; Andrade et al., 2007; Ku et al., 2009; Biemans-Oldehinkel et al., 2011; Romo-Castillo et al., 2014). This virulence-associated complex is homologous to the flagellar FliI/FliH/FliJ ATPase complex (Minamino and MacNab, 2000b; Ibuki et al., 2011). The crystal structure of the ATPase EscN has been solved, and like FliI, it shows structural similarity to the F1-ATPase α and β subunits (Imada et al., 2007; Zarivach et al., 2007). EscL, like FliH, has been evolutionarily linked to the b and δ subunits of the F1 ATPase (Pallen et al., 2005, 2006). Even more, despite the lack of sequence conservation, *in silico* and functional data suggest that EscO is the evolutionary counterpart of the flagellar FliJ protein, which in turn has been related to the γ subunit of the F1-ATPase (Ibuki et al., 2011; Romo-Castillo et al., 2014). The functional relatedness of EscO and FliJ was confirmed by heterologous complementation of the motility of a Δ*fliJ* mutant with a plasmid encoding EscO (Romo-Castillo et al., 2014). Taken together, these similarities would imply that an energy-producing enzyme, a motility machinery, and an interkingdom protein transport device, share a common evolutionary ancestor.

EscN is a peripheral membrane protein located at the base of the needle complex that energizes the secretion process, probably by releasing the substrate from its cognate chaperone and unfolding it for further secretion, as has been reported for *Salmonella* InvC, and functioning as a docking site for chaperone-substrate complexes (Gauthier and Finlay, 2003; Akeda and Galán, 2005; Thomas et al., 2005; Chen et al., 2013). This enzyme oligomerizes into a homohexameric ring structure and the oligomeric state of the protein affects its specific activity (Andrade et al., 2007; Zarivach et al., 2007). EscN interacts with EscL, a negative regulator that inhibits its ATPase activity, and with EscO, which conversely, stimulates EscN ATPase activity (Biemans-Oldehinkel et al., 2011; Romo-Castillo et al., 2014).

A model proposed for the ATPase complex function that includes previous findings in the flagellar and virulence T3S systems, suggests that the formation of the EscN-EscL complex in the cytoplasm ensures that the ATPase activity is inhibited until ATP hydrolysis can be coupled to protein secretion, preventing futile energy expenditure (Minamino and MacNab, 2000b; Blaylock et al., 2006; Stone et al., 2011). Once the EscN/EscL/EscO complex is formed near the vicinity of the export apparatus through protein-protein interactions with the C-ring, both EscL and EscO, in a similar way to their T3SS counterparts, interact with the major export gate component EscV (Zhu et al., 2002; González-Pedrajo et al., 2006; Morita-Ishihara et al., 2006; Hara et al., 2012; Cherradi et al., 2014; Lee et al., 2014), promoting a conformational change that allows EscO to stimulate EscN oligomerization and, subsequently, its ATPase activity (Claret et al., 2003; Evans et al., 2006; Ibuki et al., 2011; Romo-Castillo et al., 2014).

EscO has also been localized in the EPEC periplasm and as previously mentioned, it was found to interact with the OM ring protein EscC (Sal-Man et al., 2012a). In addition, similarly to what has been observed for FliJ, EscO interacts with the chaperones CesA2 (EscG) and CesL (Mpc) (Lin et al., 2014), suggesting that this protein performs additional roles during the T3S process. Likewise, EscL has been recently implicated as a component of the sorting platform as will be discussed next (Lara-Tejero et al., 2011; Hu et al., 2015).

#### C-ring/sorting platform

In A/E pathogens the C-ring is proposed to be a cytoplasmic annular structure located at the base of the basal body, formed mainly by the EscQ protein (formerly known as SepQ), which belongs to the YscQ/FliN (SctQ) protein family (Figure 2; Pallen et al., 2005; Biemans-Oldehinkel et al., 2011). Other members of this family are YscQ (from *Yersinia* spp.), SpaO (from *S. enterica* SPI-1), Spa33 (from *S. flexneri*), SsaQ (from *S. enterica* SPI-2), and HrcQ (from *Xanthomonas* spp.) (Morita-Ishihara et al., 2006; Lara-Tejero et al., 2011; Yu et al., 2011; Bzymek et al., 2012; Lorenz et al., 2012; Notti et al., 2015). Three different proteins, FliM, FliN, and FliG, form the corresponding flagellar C-ring, and it has been shown that members of the SctQ family have an evolutionary relationship with FliM and FliN (Zhao et al., 1996; Hueck, 1998; Pallen et al., 2005; Thomas et al., 2006). The mRNA from most of the *sctQ* homologs contains an internal alternative translation site, giving rise to a full-length protein (SctQ-Full of approximately 300 amino acids) resembling the flagellar FliM protein, and to a shorter C-terminal version (SctQ-C of ca. 100 amino acids) similar to FliN. Both translation products, SctQ-Full and SctQ-C, are required for T3S assembly and function (Bzymek et al., 2012; Notti et al., 2015; McDowell et al., 2016) with the exception of SsaQ-C from *Salmonella* SPI-2 that serves as a chaperone for SsaQ-Full but is not essential for T3S (Yu et al., 2011). To date it remains unclear whether EscQ has an internal translation start site.

The SctQ-Full and SctQ-C proteins have been shown to interact in a 1:2 ratio, and it is proposed that this complex assembles into higher order oligomers that form a ring shaped structure (Bzymek et al., 2012; Diepold et al., 2015; McDowell et al., 2016). The SctQ-Full:SctQ-C protein ratio seems to be important for proper injectisome assembly as it was shown that there is a linear correlation between the SctQ-Full structure assembly and the expression levels of SctQ-C protein (Diepold et al., 2015). The injectisome SctQ-Full/SctQ-C complex is believed to mirror the FliM/FliN basic building block of the flagellar C-ring (Bzymek et al., 2012; McDowell et al., 2016). The solved structures of several SctQ proteins revealed the presence of SpoA domains that mediate both SctQ-C-SctQ-C homotypic and SctQ-Full-SctQ-C heterotypic associations (Bzymek et al., 2012; Notti et al., 2015; McDowell et al., 2016). It was recently reported that the *Y. enterocolitica* C-ring is formed by 22 ± 8 YscQ-Full subunits per injectisome and has an estimated diameter of 30.2 nm, making it similar to that of the flagellum (Diepold et al., 2015). Furthermore, Hu and colleagues determined the structure of the *S. flexneri* C-ring by cryoelectron tomography and showed that it is formed by six pod-like structures made out of multiple copies of Spa33, arranged in a hexagonal array of 32 nm in diameter and 24 nm in height. The top portion of the pods, suggested to be composed of Spa33-Full and MxiK, links the entire C-ring to the membrane-associated components of the basal body. The bottom of each pod, proposed to be formed by a homotetramer of Spa33-C, is connected to the ATPase Spa47 (SctN) through six spoke-like densities made out of MxiN (SctL) (Hu et al., 2015). The ultrastructural details of the C-ring in A/E pathogens have not yet been addressed. In *Y. enterocolitica* it has been shown that the C-ring is a highly dynamic substructure that exchanges subunits between a YscQ cytosolic pool and the assembled structure at the base of the injectisome, and that the rate of this subunit exchange correlates with the effector secretion status (Diepold et al., 2015).

Co-immunoprecipitation experiments in EPEC revealed that EscQ interacts with the ATPase complex components EscN and EscL. Moreover, EscQ was found to be located in the cytoplasm as well as associated with the membrane and this subcellular localization was independent of its binding partners (Biemans-Oldehinkel et al., 2011). Likewise, the EscQ homologs Spa33, YscQ, and SpaO have been shown to interact with members of the SctK and SctL families (MxiK/MxiN, YscK/YscL, OrgA/OrgB; Jackson and Plano, 2000; Jouihri et al., 2003; Lara-Tejero et al., 2011). In *S. enterica*, SpaO, OrgA, and OrgB form a large molecular weight complex that interacts with translocators and effectors in a sequential order, leading to the suggestion that the C-ring may act as a sorting platform, queuing the different substrate categories to establish the correct secretion hierarchy. Both chaperones and molecular switch proteins have an active role in this selection process, acting in concert with the sorting platform to ensure an orderly secretion (Lara-Tejero et al., 2011). In *Y. enterocolitica* and *S. enterica* the formation of the C-ring/sorting platform substructure depends on the presence of both SctK (YscK and OrgA) and SctL (YscL and OrgB) proteins (Lara-Tejero et al., 2011; Diepold et al., 2015). In contrast, the sorting platform visualized from *Shigella* Δ*mxiN* minicells showed that structural pods are assembled even in absence of the MxiN (SctL) protein (Hu et al., 2015). Based on the information for the homologous proteins, in A/E pathogens the sorting platform might be formed by EscQ, EscL, and EscK (formerly Orf4), a component that has been associated with the SctK protein family (Abby and Rocha, 2012; Barison et al., 2013). Like other members of the SctK protein family such as OrgA from *Salmonella* and MxiK from *Shigella*, EscK is critical for T3 secretion (Sukhan et al., 2001; Jouihri et al., 2003; Deng et al., 2004). The precise composition and function of the sorting platform in A/E pathogens remains to be investigated.

#### Effectors and chaperones

The ultimate goal of T3SS assembly is to modulate host cell functions for the benefit of the bacterium, and this is accomplished by the interplay of the biochemical activities displayed by injected effectors. A/E pathogens share seven effectors encoded in the LEE-PAI, and additionally each pathogen has its own suite of effectors encoded outside the LEE, termed non-LEE-encoded (Nle) effectors, which are encoded on prophages or other integrative elements (McDaniel et al., 1995; Deng et al., 2003, 2004, 2010; Tobe et al., 2006; Iguchi et al., 2009).

The LEE-encoded effectors are Tir, Map, EspF, EspG, EspH, EspZ, and EspB (Garmendia et al., 2005). It has been shown in EPEC that LEE-encoded effectors are hierarchically secreted/translocated into host cells, being Tir the first effector to be injected followed by EspZ, EspF, EspH, EspG, and Map (Thomas et al., 2007; Mills et al., 2008). As previously mentioned, although EspB is a structural translocon component, it is also translocated into host cells but in much lower levels compared to the other effectors, thus precluding a meaningful analysis of its translocation hierarchy (Wolff et al., 1998; Mills et al., 2008). The total number of effectors varies between pathogens, *C. rodentium* possesses a reported repertoire of at least 29 effectors, while EPEC E2348/69 encodes ca. 22 and EHEC O157:H7 encodes 39 (Tobe et al., 2006; Dean and Kenny, 2009; Petty et al., 2010). Some of the Nle effectors, like NleA/EspI, NleB, NleE, NleF, NleG, NleH, EspJ, and EspL, are common to all LEE-encoding species sequenced to date (Petty et al., 2010). A more recent hierarchical translocation order was reported including both LEE and Nle effectors, in which Tir is again the first one to be injected followed by EspZ, NleA, NleH1, EspF, EspH, NleH2, EspJ, Map, EspG, NleD, NleF, NleB1, NleE1, NleB2, NleC, NleG, NleE2, EspG2, and EspL2 (Mills et al., 2013). For a detailed description of the specific function of effectors in A/E pathogens, we refer to reviews on the subject (Dean and Kenny, 2009; Jayamani and Mylonakis, 2014; Santos and Finlay, 2015).

In general, effectors contain one or more C-terminal catalytic domains that manipulate host cell functions, while their N-termini mediates chaperone recognition and transport via the T3SS (Ghosh, 2004). The first ca. 20 amino acids contain the secretion signal sequence necessary and sufficient for T3 secretion (Crawford and Kaper, 2002; Munera et al., 2010; Deng et al., 2015). The chaperone-binding domain is located downstream of the N-terminal secretion signal, usually between amino acids 50 and 100 (Blocker et al., 2003). This domain adopts a linear conformation and wraps around the chaperone dimer through a β-motif (Lilic et al., 2006).

The T3SS-associated chaperones are small and acidic cytoplasmic proteins important for efficient secretion of their cognate substrates (Feldman and Cornelis, 2003). They promote T3 substrate secretion by contributing to protein stability, aiding in substrate recognition and targeting, preventing premature oligomerization and unspecific interactions, and participating in the establishment of a secretion hierarchy (Wainwright and Kaper, 1998; Abe et al., 1999; Elliott et al., 1999a; Gauthier and Finlay, 2003; Neves et al., 2003; Parsot et al., 2003; Creasey et al., 2003a,c; Thomas et al., 2005, 2007; Su et al., 2008; Chen et al., 2013; Sal-Man et al., 2013; Allison et al., 2014). T3 chaperones function in an ATP-independent manner, although chaperone dissociation from the substrate-chaperone complex is an ATP-dependent process (Akeda and Galán, 2005). These proteins have been grouped into three classes depending on their binding substrates: class I chaperones that bind effectors (class IA, one effector and class IB, multiple effectors), class II chaperones that bind translocators, and class III chaperones that bind the needle component (Cornelis and Van Gijsegem, 2000; Page and Parsot, 2002; Parsot et al., 2003; Cornelis, 2006).

The LEE encodes eight proteins that have been characterized as chaperones: CesF, CesL, CesT, CesAB, CesD, CesD2, EscE, and EscG/CesA2 (Wainwright and Kaper, 1998; Elliott et al., 1999a, 2002; Luo et al., 2001; Creasey et al., 2003a,c; Neves et al., 2003; Thomas et al., 2005; Younis et al., 2010; Ramu et al., 2013; Sal-Man et al., 2013). These proteins, with the exception of CesF and CesD2, are critical for *C. rodentium* virulence in mice (Deng et al., 2004). CesT was originally classified as a class IA chaperone for the Tir effector, but subsequent reports showed that it interacts with 10 additional effectors: Map, NleA, NleF, NleH, NleH2, EspH, EspZ, EspF, NleG, and EspG, being re-classified as a multicargo class IB chaperone (Abe et al., 1999; Elliott et al., 1999a; Creasey et al., 2003a,b; Thomas et al., 2005, 2007; Mills et al., 2008). CesF is a class IA chaperone that binds EspF (Elliott et al., 2002) and is required for its translocation (Mills et al., 2008). Additionally, based on sequence similarity searches, CesL was recently classified as a class I chaperone for SepL although it is a special case, since its substrate seems to be an aberrant effector that is not secreted (Younis et al., 2010).

Four class II chaperones have been described in A/E pathogens: CesD, CesD2, CesAB, and CesA2. CesD assists EspD and EspB secretion, although it has only been shown to directly interact with EspD (Wainwright and Kaper, 1998), which has an additional chaperone, CesD2 (Neves et al., 2003). The CesAB chaperone differs from the others in that it has a basic pI. It interacts specifically with EspA and EspB, preventing their polymerization, maintaining them partially unfolded and promoting their stabilization (Creasey et al., 2003b; Yip et al., 2005b). Finally, CesA2 was identified in EHEC as a second chaperone for EspA (Su et al., 2008). However, it was later demonstrated in EPEC that CesA2 is a class III chaperone that binds to the needle subunit EscF and therefore, it was named EscG, which together with EscE avoid premature polymerization of the needle (Sal-Man et al., 2013).

## Secretion regulation

**Figure 4:**
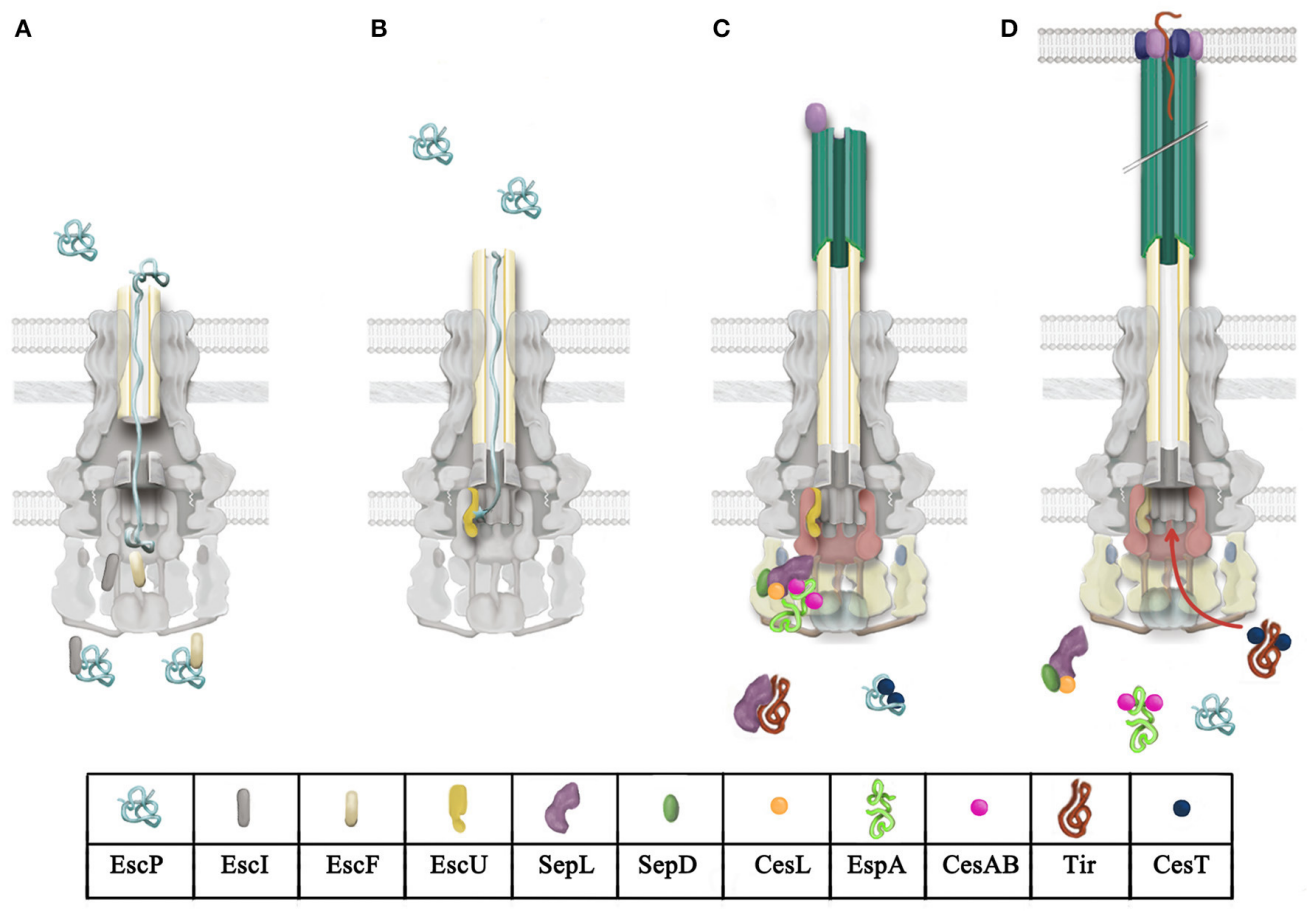
**Model for T3 secretion regulation in A/E pathogens**. **(A)** Rod and needle assembly occur simultaneously (Marlovits et al., 2006; Lefebre and Galán, 2014). EscP directly interacts with early substrates (EscI and EscF, rod and needle subunits, respectively), regulating its secretion. EscP is secreted occasionally during needle assembly. **(B)** Once rod and needle assembly is completed, EscP makes contact with the full-length needle, causing a pause in substrate secretion that allows the productive interaction between EscP and the pre-cleaved C-terminal domain of EscU (EscUcc) (Monjarás Feria et al., 2012). This interaction is proposed to promote a conformational change in EscUcc that flicks substrate specificity, probably generating a docking site for a different category of substrates (Zarivach et al., 2008; Thomassin et al., 2011). **(C)** Translocator secretion is now allowed. The SepL/SepD/CesL complex targets translocator/chaperone complexes to the sorting platform (formed by EscQ, EscL, and EscK; Lara-Tejero et al., 2011). SepL also interacts with the export gate component EscV and probably modifies its affinity for certain substrate classes; it has also been proposed that it might block access of effectors to the export gate (Lee et al., 2014; Shen and Blocker, 2016). SepL interacts with the effector Tir, preventing its secretion (Wang et al., 2008). **(D)** Upon host cell contact, the SepL/SepD/CesL complex might disengage from the export gate component and the sorting platform, alleviating the effector recognition blockade exerted on EscV and allowing effector translocation into host cells.

Efficient assembly and function of the T3SS depend on the hierarchical and temporal control of substrate secretion. The assembly of the injectisome is a sequential process that initiates with a Sec-dependent secretion stage in which the OM and IM ring proteins, as well as the export apparatus components, are integrated into the bacterial membranes, then, the ATPase complex and C-ring constituents associate with the basal body to form a functional T3SS (Diepold et al., 2010; Wagner et al., 2010a). Subsequently, the remaining components are secreted in a T3SS-dependent and ordered manner. T3 substrates are classified into three categories according to their temporal secretion: early (inner rod and needle), middle (translocators), and late (effectors) substrates. The hierarchical secretion of these proteins is regulated by two substrate specificity-switching mechanisms that involve protein complexes referred to as molecular switches (Tree et al., 2009; Deane et al., 2010; Büttner, 2012). The secretion regulation mechanisms are reviewed below and a working model for A/E pathogens, based on recent studies in the field, is illustrated in Figure 4.

### Molecular switch 1: EscP/EscU

The transition from early to middle and late substrate secretion occurs once the needle reaches its proper length, a mechanism regulated, as mentioned above, by T3S4 proteins (Minamino et al., 2004; Büttner, 2012), such as EscP from EPEC, YscP from *Yersinia*, InvJ from *Salmonella*, Spa32 from *Shigella*, HpaC from *Xanthomonas* and FliK in the flagellar T3SS (Kubori et al., 2000; Magdalena et al., 2002; Tamano et al., 2002; Journet et al., 2003; Minamino et al., 2004; Lorenz et al., 2008; Monjarás Feria et al., 2012). Most T3S4 proteins are secreted, and its absence results in the assembly of abnormally long needle/hook structures (Hirano et al., 1994; Kawagishi et al., 1996; Minamino et al., 1999; Payne and Straley, 1999; Kubori et al., 2000; Stainier et al., 2000; Magdalena et al., 2002; Tamano et al., 2002; Agrain et al., 2005; Waters et al., 2007; Monjarás Feria et al., 2012). Interestingly, HpaC, the only non-secreted T3S4 protein is not involved in pili length regulation (Büttner et al., 2006). The absence of YscP, Spa32, HpaC, and FliK also leads to a reduction or complete absence of middle and/or late substrate secretion, suggesting that besides controlling needle/hook length, T3S4 proteins also promote the secretion substrate specificity switch (Hirano et al., 1994; Kawagishi et al., 1996; Williams et al., 1996; Minamino et al., 1999; Stainier et al., 2000; Magdalena et al., 2002; Tamano et al., 2002; Edqvist et al., 2003; Journet et al., 2003; Botteaux et al., 2008; Morris et al., 2010; Lorenz and Büttner, 2011; Schulz and Büttner, 2011). In EPEC, however, elimination of *escP* results in a reduced secretion of middle substrates but enhanced secretion of effectors, this led to the proposal that EscP is not indispensable for substrate switching, although it increases the efficiency of the switching event (Monjarás Feria et al., 2012). In agreement, a Δ*spa32* mutant can still form translocation pores and display some RBCs hemolytic activity, indicating that it autonomously switches substrate specificity (Shen et al., 2012).

Substrate switching is regulated by the interaction of T3S4 proteins with members of the SctU export apparatus protein family. T3S4 proteins interact with the C-terminal domain of SctU, as shown for EscP/EscU, Spa32/Spa40, HpaC/HrcU, and FliK/FlhB (Minamino and Macnab, 2000a; Botteaux et al., 2008; Lorenz et al., 2008; Morris et al., 2010; Monjarás Feria et al., 2012). This interaction is proposed to induce conformational changes in SctU proteins that in turn change their specificity of substrate secretion, regulating the switching event. The finding that extragenic suppressor mutations of *fliK* and *yscP* are localized in the C-terminal domains of YscU and FlhB, respectively, supports this idea (Kutsukake et al., 1994; Williams et al., 1996; Edqvist et al., 2003).

The YscU/FlhB protein family undergoes spontaneous autocleavage in the NPTH motif found in an exposed region at its C-terminal domain. The two resulting cleavage products remain tightly associated, as has been demonstrated for EscU in EPEC, YscU in *Yersinia*, Spa40 in *Shigella* and FlhB in the flagellar T3SS (Minamino and Macnab, 2000a; Deane et al., 2008a; Björnfot et al., 2009; Monjarás Feria et al., 2012). The autoproteolytic event changes the orientation of the PTH loop and is proposed to create a potential interaction surface for other T3SS proteins (Lavander et al., 2002; Ferris et al., 2005; Deane et al., 2008a; Zarivach et al., 2008; Björnfot et al., 2009; Lountos et al., 2009; Wiesand et al., 2009). Since non-cleavable mutants reduce secretion of intermediate and late substrates, it is thought that a docking surface for different T3S substrates might be created upon proteolytic cleavage of SctU proteins (Fraser et al., 2003; Sorg et al., 2007; Zarivach et al., 2008; Lorenz and Büttner, 2011; Thomassin et al., 2011). In EPEC, a subtle conformational change in a conserved surface was observed in non-cleavable mutants (Zarivach et al., 2008). Besides this change in structure, in a non-cleavable EscU mutant background, the membrane association of the multicargo chaperone CesT is reduced, suggesting that the cleavage is important for substrate docking; however, no direct interaction between CesT and EscU could be demonstrated (Thomassin et al., 2011). In any case, although indispensable for secretion regulation, the cleavage event *per se* is not the signal that triggers the substrate secretion change; instead, it might allow YscU/FlhB protein family members to acquire the conformation required for its substrate switching function (Minamino and Macnab, 2000a; Zarivach et al., 2008; Monjarás Feria et al., 2015).

The molecular mechanisms that regulate needle/hook length and the secretion substrate specificity switch have been extensively studied in various systems, leading to the proposal of different models that will be discussed below.

#### Single ruler model

The observation that the size of YscP correlates with the length of the needle, led Journet and colleagues to propose that this protein functions as a molecular ruler that directly measures needle length (Journet et al., 2003). In this static ruler model, YscP is positioned within the inner channel of the growing needle, with its N- and C-terminal domains probably contacting the needle tip and the T3SS base, respectively, allowing the secretion of needle subunits. When the molecular ruler is completely stretched, it interacts with YscU at the T3SS base to switch substrate secretion, and gets secreted, finishing the export of needle subunits and permitting middle and late substrate secretion (Journet et al., 2003). Afterwards it was demonstrated that only one YscP molecule was required for length control and that, while measuring needle length, it preserved its helical structure (Wagner et al., 2009, 2010b). However, since the diameter of an α-helix is approximately 10 Å and that of the secretion channel is 13 Å, it is impossible to fit a ruler protein and a secreted needle subunit in it (Fujii et al., 2012). Under these same physical constraints, the simultaneous secretion of FliK and hook subunits in the flagellar system was also suggested to be improbable, and therefore an alternative model was proposed for hook length regulation (Shaikh et al., 2005; Moriya et al., 2006).

#### Infrequent ruler model

In agreement with the previous model, FliK, Spa32, and InvJ have also been demonstrated to act as molecular rulers (Shibata et al., 2007; Botteaux et al., 2008; Erhardt et al., 2011; Wee and Hughes, 2015). However, the proposed needle/hook measurement mechanisms are different. The alternative dynamic-ruler model was originally proposed in the flagellar system by Moriya et al. (2006). This model suggests that during hook assembly FliK is occasionally exported, and that when found in the central channel secretion is briefly paused, allowing the measurement of hook length through the temporary interaction of the N- and C-terminal domains of FliK with the hook cap FlgD and FlhB, respectively. Short hooks do not allow productive interactions with FliK, inducing its rapid secretion, however, as the hook assembles, more frequent interactions between FliK and the growing structure occur, slowing the secretion rate. Finally, when the hook length reaches approximately 55 nm, the slow secretion rate enables the completely stretched FliK to successfully interact with FlhB, which flips the switch in substrate secretion (Erhardt et al., 2010a, 2011). A similar mechanism was proposed for the needle length control in EPEC. EscP, which directly interacts with EscF, measures needle length upon intermittent secretion during needle assembly (Figure 4A). When this structure reaches its final length, all subdomains of EscP can make contact with the needle, promoting a pause in secretion that allows EscP to interact with EscU, inducing a conformational change that modifies its specificity from early to middle and late substrates (Figure 4B; Monjarás Feria et al., 2012).

#### Role of the inner rod in needle length and substrate switching

An alternative model was proposed for the SPI-1 injectisome in *S. enterica*, which states that the assembly of the inner rod determines the size of the needle. Therefore, the substrate switching is the result of conformational changes produced at the base of the T3SS in response to inner rod assembly completion (Marlovits et al., 2006). Many pieces of evidence support this model. An *invJ* null mutant, which assembles long needles (Kubori et al., 2000), secretes high levels of the inner rod subunit PrgJ, but fails to assemble this structure, thus, InvJ was proposed to promote inner rod assembly. In addition, the overexpression of PrgJ results in the assembly of shorter needles, apparently because of faster inner rod assembly (Sukhan et al., 2003; Marlovits et al., 2006). Moreover, mutations in PrgJ that slowed the rate of rod assembly, presumably due to impaired subunit-subunit interactions, lead to the assembly of longer needles (Lefebre and Galán, 2014). Likewise, a role of the inner rod component in substrate specificity switching has been proposed in *Y. pseudotuberculosis*. It was shown that the inner rod protein YscI is hypersecreted in a *yscP* mutant background and that a suppressor mutation in YscU reestablishes normal YscI secretion, suggesting that the inner rod assembly is important for the substrate specificity switching mechanism (Wood et al., 2008). In EPEC, EscP was shown to directly interact with the inner rod subunit EscI, and it is proposed to regulate its secretion, since an *escP* null mutant hypersecretes EscI (Monjarás Feria et al., 2012). Besides, EscI has also been shown to interact with the export gate protein EscU (Creasey et al., 2003b; Sal-Man et al., 2012b).

It is possible that a combination of these models will contribute to the molecular mechanism of needle length control and substrate specificity switching. Finally, the outcome of the first molecular switch is the assembly of a proper size needle and the trigger of middle and late substrate secretion.

### Molecular switch 2: SepL/SepD

The second molecular switch regulates differentially the secretion of translocators and effectors. A family of proteins known as gatekeepers participates in this switch. It comprises proteins like SepL from A/E pathogens (Kresse et al., 2000; Deng et al., 2004; O'Connell et al., 2004), MxiC from *Shigella* (Botteaux et al., 2009), InvE and SsaL from *Salmonella* SPI-1 and SPI-2, respectively (Kubori and Galán, 2002; Coombes et al., 2004), CopN from *Chlamydia* (Fields and Hackstadt, 2000), YopN/TyeA from *Yersinia* (Forsberg et al., 1991; Iriarte et al., 1998), and PopN/Pcr1 from *Pseudomonas* (Yang et al., 2007). In the latter two cases, the protein is divided in two polypeptide chains that correspond to the N- and C-terminal domains of the full-length proteins.

In A/E pathogens, elimination of SepL abolishes secretion of the translocators and increases secretion of effectors (Deng et al., 2004; O'Connell et al., 2004; Wang et al., 2008). This phenotype is identical to that of the *ssaL* mutant in the *Salmonella* SPI-2 (Coombes et al., 2004; Yu et al., 2010). In the case of MxiC and InvE, their absence results in a decreased secretion of translocators and increased secretion of effectors (Kubori and Galán, 2002; Botteaux et al., 2009; Martinez-Argudo and Blocker, 2010), while elimination of YopN leads to a constitutive secretion of both translocators and effectors (Forsberg et al., 1991; Day and Plano, 1998). These mutant phenotypes suggest that these proteins are bifunctional, promoting translocator secretion (with exception of YopN) and avoiding the premature secretion of effectors (Figure 4C). In EPEC and EHEC SepL interacts with SepD, whose deletion in A/E pathogens results in the same phenotype as the *sepL* null mutant (Deng et al., 2004; O'Connell et al., 2004). Aside from its role in secretion regulation, no further information has been published on SepD function.

#### Promoting translocator secretion

The mechanism of translocator secretion regulation is not completely understood; however, in *Salmonella* it was shown that InvE directly interacts with the chaperone/translocator complex, though not with its individual components (Kubori and Galán, 2002). Additionally, the Chlamydial gatekeeper CopN interacts with the translocator-specific chaperone Scc3, and this interaction is important not only for translocator secretion but also for timely secretion prior to effectors (Archuleta and Spiller, 2014). Likewise, *Shigella* MxiC was shown to interact with the translocator chaperone IpgC (Cherradi et al., 2013). These results highlight the importance of the interaction of the gatekeeper with the translocator/chaperone complex or its individual components in the establishment of the secretion hierarchy. Furthermore, as aforementioned, in a seminal work Lara-Tejero et al. demonstrated the existence of a sorting platform that selects substrates for secretion. Translocators are loaded into the sorting platform in an InvE dependent manner, and elimination of translocators or the gatekeeper results instead in the loading of effectors (Lara-Tejero et al., 2011).

Recently, the crystal structure of SepL from EPEC was determined (Burkinshaw et al., 2015b). It showed that despite the low sequence similarity between gatekeeper proteins, its overall structure is well conserved, with a general architecture consisting of three helical X-bundle domains. When compared to the solved structures of CopN, YopN/TyeA and MxiC (Schubot et al., 2005; Deane et al., 2008b; Archuleta and Spiller, 2014), the SepL structure showed that among the regions conserved between these proteins one corresponds to the binding site of the translocator chaperone Scc3 in CopN. Protein sequence analysis highlighted the presence of three highly conserved residues: Tyr188, Phe327 and Arg333 (Burkinshaw et al., 2015b). In CopN, Arg365 (equivalent to Arg333 in SepL) is essential for the CopN/Scc3 interaction (Archuleta and Spiller, 2014). In contrast, alanine substitution of Arg333 in SepL did not affect the secretion phenotype (Burkinshaw et al., 2015b), though an interaction between SepL and translocators or their chaperones in A/E pathogens has yet to be demonstrated. In conclusion, the precise molecular mechanism through which gatekeepers promote translocator secretion remains an open question.

#### Preventing premature secretion of effectors

The prevention of effector secretion before host cell contact is a common feature of all gatekeeper proteins. For some of them, this regulatory function relies in their ability to be secreted, e.g., YopN, MxiC, CopN, and PopN (Day and Plano, 1998; Fields and Hackstadt, 2000; Yang et al., 2007; Botteaux et al., 2009). In the case of *Yersinia*, YopN functions like a plug that physically blocks the secretion channel while being attached to the T3SS base through its interaction with TyeA, however, in response to low calcium concentration, this interaction is disrupted, YopN is secreted and the blockade is relieved allowing the secretion of Yops (Cheng et al., 2001). In *Chlamydia*, the secretion of CopN is also essential for secretion of effectors (Archuleta and Spiller, 2014; Shen et al., 2015). Additionally, a recent model of effector secretion control in *Pseudomonas* suggests that the PopN complex (PopN, Pcr1, PscB, and Pcr2) is tethered to the T3S apparatus via the Pcr1/PcrD (SepL C-terminal domain/EscV) interaction, blocking the access of effectors to the secretion apparatus. However, when the appropriate signal for effector secretion is received, PopN is secreted, permitting the loading of effectors onto the sorting platform and its subsequent docking to the export gate for secretion (Lee et al., 2014). A similar mechanism was proposed for MxiC from *Shigella*, in which its interaction with the inner rod protein MxiI, blocks access of effectors to the entry gate. Nevertheless, since this mechanism is also conserved for InvE in *Salmonella*, which is not secreted, its regulation might rely instead in the ability of MxiC to engage with the rod and not in its secretion capacity (Botteaux et al., 2009; Cherradi et al., 2013).

In A/E pathogens, neither SepL nor SepD have been observed to be secreted. Therefore, a different secretion regulation mechanism was proposed in EHEC, in which SepL was shown to bind to the effector Tir. It was demonstrated that while translocators are secreted, the interaction of Tir with SepL delays its secretion, avoiding the premature export of all other effectors, which suggests that this interaction controls the proper timing of secretion (Figure 4C; Wang et al., 2008). This mechanism is supported by the demonstration that Tir secretion is important for the secretion of the rest of the effectors (Thomas et al., 2007). In addition, as aforementioned, there is an established hierarchy in the secretion of effectors in which Tir is the first effector to be secreted (Figure 4D; Mills et al., 2008, 2013). In this regard, it has also been shown that although Tir translocation is not essential for the translocation of other effectors, its absence negatively influences their translocation efficiency (Mills et al., 2013). Finally, it was suggested that different interacting partners of SepL are required for its secretion regulatory function. This is sustained by the fact that the same region of SepL that is required for Tir binding is also essential for its interaction with EscD (Wang et al., 2008).

Even though SepL is not secreted, it was proposed to be an aberrant effector because when fused to a reporter protein, its N-terminal domain is able to mediate protein secretion. Moreover, the SepL-SepD complex was shown to interact with a third protein named CesL, which like SepD, is suggested to function as a SepL chaperone (Younis et al., 2010). The SepL-SepD-CesL complex resembles the YopN/TyeA-YscB-SycN complex in *Yersinia* (Day and Plano, 1998), PopN/Pcr1-PscB-Pcr2 complex in *Pseudomonas* (Yang et al., 2007), CopN-Scc4-Scc1complex in *Chlamydia* (Silva-Herzog et al., 2011) and SsaL-SpiC-SsaM complex in *Salmonella* SPI-2 T3SS (Yu et al., 2010). An outstanding work by Yu and colleagues demonstrated that the SsaL-SpiC-SsaM complex regulates the secretion hierarchy in response to pH changes. This protein complex associates with the T3SS base under the acidic conditions of the *Salmonella*-containing vacuole, allowing the secretion of translocators while blocking the secretion of effectors. When the translocation pore assembles in the vacuolar membrane, an increase in pH is detected and transmitted to the T3SS base, which in turn causes dissociation of the SsaL-SpiC-SsaM complex and the degradation of its individual components, permitting the secretion of effectors (Yu et al., 2010). In A/E pathogens it has been shown that, upon removal of calcium from the growth medium, translocator secretion is reduced while effector secretion is increased, a phenotype that partially mimics that of the *sepL* or *sepD* mutants (Kenny et al., 1997a; Ide et al., 2003; Deng et al., 2005). This suggests that the absence of calcium might be the environmental cue that regulates the secretion hierarchy, probably through the SepL/SepD complex (Deng et al., 2005).

## Concluding remarks

Enteropathogenic and enterohemorrhagic *E. coli* are an important cause of gastroenteric disease worldwide. These *E. coli* pathotypes remain as a major public health concern both in the third world population, where EPEC infections predominate, and in developed countries where EHEC is responsible for food-borne outbreaks. The T3SS is an essential virulence trait in the pathogenesis of these bacteria, thus, it is crucial to understand the mechanistic and functional characteristics of this complex machinery. Remarkable advances have been made in the T3SS field in A/E pathogens. To date, almost all of the genes encoded in the LEE PAI have an assigned function, with *rorf1* being the exception. Nevertheless, the function of some proteins, such as EscK, has only been deduced based on similarities shared with proteins of other T3S systems. Hence, to have a complete understanding of the injectisome, we must expand our efforts to uncover the precise role of such unexplored components. Moreover, numerous crystal structures of A/E pathogens T3SS components have been solved, contributing to a deeper understanding of protein function and interactions, as well as of the overall architecture and assembly of the injectisome. However, there are still several structural and mechanistic details that need to be elucidated, such as determining the precise composition and function of the export apparatus and cytoplasmic components and unraveling the mechanism of protein transport through a 600 nm filament. In addition, *C. rodentium* has proved to be an invaluable tool for *in vivo* analyses of T3SS function. Nonetheless, cheaper and more practical invertebrate models such as *Galleria mellonella* are emerging to understand the virulence mechanisms of A/E pathogens (Leuko and Raivio, 2012). T3S regulation has also been studied in A/E pathogens, showing that highly conserved mechanisms involving two molecular switches and a sorting platform act in coordination to establish a strict hierarchy of substrate secretion. Yet, the physiological signal that triggers effector translocation upon host cell contact is still unknown.

Recent advances in fluorescence microscopy, cryo-electron tomography and single molecule super-resolution techniques have greatly contributed to deciphering the architecture of this complex machine. The use of these tools showed that the T3S systems are not static structures, but instead are dynamic molecular machines that undergo several changes to adapt to different secretion states; for example, employing FRAP (fluorescence recovery after photobleaching), the C-ring main component was demonstrated to exchange between a cytoplasmic and an assembled state (Diepold et al., 2015). In addition, by performing TIRF-FRAP (total internal reflection fluorescence and FRAP) experiments, the ATPase complex was proposed to function as both, a dynamic substrate carrier and a static substrate loader (Bai et al., 2014). Moreover, cryo-electron tomography revealed that these cytoplasmic complexes are structurally stabilized, and the basal body adopts a compacted conformation, upon host cell contact (Nans et al., 2015). Thus, these real time experiments in A/E pathogens are required to disclose the dynamic nature of the T3SS in action. Despite all this knowledge, there are multiple open questions that remain elusive, e.g., the signaling cascade from host cell sensing to signal transduction and response of the cytoplasmic components, substrate targeting and docking and the energizing of the secretion process. An intriguing issue is how the T3SS basal machinery recognizes the secretion signal of T3 substrates and which is the precise interaction path that substrate-chaperone complexes have to follow. Likewise, although it has been proposed that the proton motive force is the main energy source for protein transport (Minamino et al., 2011; Lee et al., 2014), the components involved in proton flow coupling to protein secretion are still unknown. The *in vitro* reconstitution of the T3SS could open up possibilities to solve these problems.

The comprehensive understanding of the structure and function of this complex secretion nanomachine will help to elucidate a way to interfere with this system, preventing bacterial pathogenicity. Drug discovery efforts to inhibit T3SS-mediated virulence in A/E pathogens must be guided by new rational approaches such as molecular docking, design of synthetic peptides and high-throughput screenings (Pan et al., 2007; Larzábal et al., 2010; Kimura et al., 2011; Duncan et al., 2012). Recently, the controlled expression of EPEC injectisomes in a non-pathogenic *E. coli* K-12 strain (Ruano-Gallego et al., 2015), showed the feasibility and the potential of using the T3SS of A/E pathogens as a molecular tool for biotechnological applications and therapeutic purposes. Finally, since poverty, malnutrition and enteric diseases are closely linked, measures to ensure access to clean water sources and basic sanitation services in susceptible communities must be guaranteed.

## Author contributions

All authors listed, have made substantial, direct and intellectual contribution to the work, and approved it for publication.

### Conflict of interest statement

The authors declare that the research was conducted in the absence of any commercial or financial relationships that could be construed as a potential conflict of interest.
